# Process Regulation of Microbial-Driven Aldehyde Metabolism in Sauce-Flavor Baijiu Fermentation

**DOI:** 10.3390/foods15010017

**Published:** 2025-12-21

**Authors:** Bo Chen, Wei Cheng, Yiyun Li, Ying Yang, Jixiang Hu, Huibo Luo, Dan Huang, Wenhua Tong, Yadong Zhang

**Affiliations:** 1 Sichuan Langjiu Co., Ltd., Luzhou 646523, China; bbovo99@163.com (B.C.); squan_wu@163.com (Y.L.); 2 School of Food and Liquor Engineering, Sichuan University of Science and Engineering, Yibin 644005, Chinasusehbluo@163.com (H.L.);; 3 Brewing Science and Technology Key Laboratory of Sichuan Province, Sichuan University of Science and Engineering, Yibin 644005, China

**Keywords:** aldehyde, flavor, microorganisms, sauce-flavor baijiu

## Abstract

Aldehyde compounds are crucial for the flavor profile of Baijiu; however, their metabolic interplay with microbial communities remains inadequately understood. This study demonstrates that the dynamics of aldehydes during Sauce-Flavor Baijiu (SFB) fermentation are primarily driven by stage-specific microbial activities. Based on microbial succession patterns, the fermentation process was divided into early (0–7 days) and late (20–30 days) stages. Seven major aldehydes were identified, with furfural being the dominant component, accounting for over 70% of the total aldehyde content. An integrated Environmental–Microbe–Flavor analysis systematically revealed stage-dependent microbial-metabolite interactions. In the early stage, dominant microorganisms such as *Fusarium* and *Apiotrichum scarabaeorum* consumed substrates including starch and reducing sugars. Their growth was strongly promoted by increasing ethanol levels and temperature, thereby accelerating aldehyde transformation. As fermentation progressed, moisture emerged as a key regulatory factor, showing a significant negative correlation with *Paenibacillus*, suggesting that moisture may shape aldehyde metabolism by modulating microbial community structure. Further moisture regulation (51.1–52.7%) applied in the seventh fermentation cycle showed that increased moisture in Zaopei was inversely correlated with aldehyde transformation efficiency (88.3–75.5%). This study elucidates the moisture-mediated regulatory mechanism underlying microbial metabolism and aldehyde conversion, offering novel insights for optimizing the fermentation process of SFB.

## 1. Introduction

Baijiu, deeply rooted in China, boasts a brewing history spanning over a thousand years. Its profound cultural heritage, coupled with intricate and diverse production techniques, has rightfully earned it a place among the six renowned distilled spirits globally [1,2]. Baijiu can be categorized into 12 distinct types, taking into account variations in raw materials, production procedures, fermentation vessels, and fermentation agents. One of these is Sauce-Flavor Baijiu (SFB) [3,4]. SFB stands out for its refined aroma, full-bodied taste, and unique brewing process. The development of its characteristic sauce-like aroma is the result of intricate biochemical processes, emerging from the interplay between fermentation techniques and microbial activities [5].

**Figure 1:**
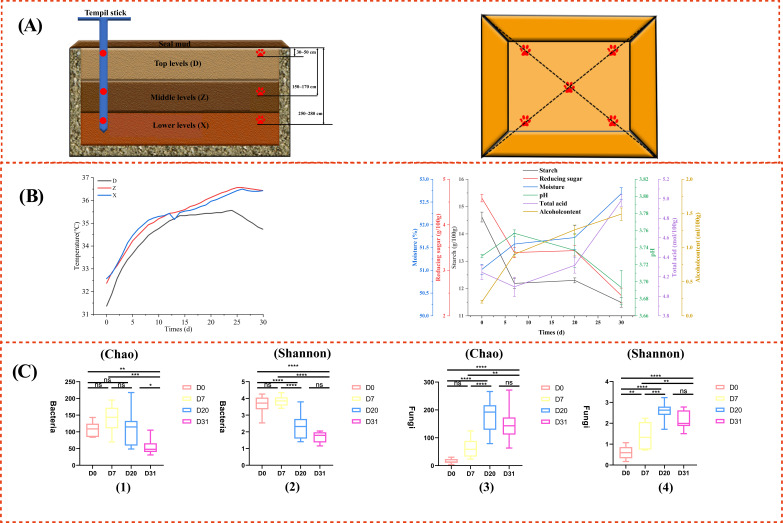
Fermented grains in the pit: (**A**) Schematic diagram of sampling points. The left side is a sectional view, and the right side is a top view. The paw-shaped symbols are the sampling points (The red graph represents the sampling marker points; D, Z, and X represent the upper, middle, and lower layers, respectively). (**B**) Dynamic changes in physicochemical properties during the fermentation process in the pit. The left figure shows the temperatures of the upper layer (black), middle layer (red) and lower layer (blue) in the pit, and the right figure shows the changes in physicochemical factors in the pit. Among them, red represents reducing sugar; black represents starch; blue represents water; green represents pH value; purple represents total acid. (**C**) Changes in the α-diversity index of the microbial community during the fermentation in the pit: (1) bacterial Chao1 index, (2) bacterial Shannon index, (3) fungal Chao1 index, (4) fungal Shannon index. Notes. ns *p* > 0.05, * *p* < 0.05, ** *p* < 0.01, *** *p* < 0.001, **** *p* < 0.0001.

The production of SFB follows a typical solid-state fermentation process [6]. The production cycle of this type of Baijiu typically spans one year. It commences around the Duanwu Festival (Dragon Boat Festival) with the production of the fermentation starter (Daqu [7]). The production of SFB is characterized by “four highs and two longs”. “Four highs” denote high temperature during Daqu production, high temperature for saccharification and stacking, high temperature for fermentation, and high temperature for distillation [8,9]. “Two longs” refer to a long production cycle (usually one year) and a long aging time (the base Baijiu is generally aged for at least three years or even longer) [10,11]. Evidently, the quality of SFB is determined by multiple factors. Pit fermentation (Figure 1A) is one of the most crucial stages in Baijiu production, directly influencing the quality of the original Baijiu. Through an investigation into the correlation among microorganisms, physicochemical properties, and flavors during the Xiasha brewing cycle, Ren Tingting et al. [12] discovered that the stacking stage enriches the microbial community essential for flavor formation, and the accumulation of flavor substances is concentrated in the pit stage [13].

Flavor is a decisive factor in determining the quality of Baijiu. Flavor analysis indicates that ethanol and moisture make up around 98% of the total mass of Baijiu, while flavor compounds account for the remaining 2% [14,15]. As research into the diverse aroma and taste compounds in Baijiu progresses, the minor components in Baijiu are gradually coming to light. At present, over 2000 substances have been identified in SFB, including esters, alcohols, acids, and aldehydes [16]. Among these, more than 300 compounds contribute to the flavor of Baijiu. Aldehyde compounds, as one of the major flavor substances in SFB, are pivotal in the formation of the sauce-aroma flavor. They can impart characteristic aromas like fruity, floral, and caramel notes, thus being an essential part of the sauce-aroma profile [17]. However, an excessive amount of aldehydes, especially Low-molecular-weight aldehydes, can produce unpleasant pungent and irritating odors, disturbing the aroma balance [18,19]. For instance, furfural is a key flavor component in liquor, playing a significant role in its unique taste and aroma [20]. As a biomass [21], furfural can also serve as an energy source for microorganisms. Benzaldehyde, an aromatic aldehyde with an active carbonyl group, ranks second only to vanillin in the global fragrance market [22]. Nevertheless, long-term or excessive consumption of these aldehydes may have toxic impacts on the human central nervous system and liver [23]. Furthermore, it has been reported that most aldehydes are intermediates of the Maillard reaction [24], which occurs during the aging process of Baijiu and influences its flavor [25]. However, the dynamic changes in aldehyde compounds during the pit fermentation process of SFB, as well as their relationship with microbial activities, remain insufficiently understood. This limitation restricts a more in-depth comprehension of the sauce-aroma flavor formation process. In recent years, emerging omics techniques such as metabolomics and high-throughput sequencing have been extensively applied in the analysis of complex fermentation systems. These techniques offer powerful tools for uncovering the interaction mechanisms between microorganisms and metabolites.

Therefore, it is necessary to reveal the microbial community succession, changes in physicochemical parameters, aldehyde metabolism, and the regulatory mechanisms among the three during the pit fermentation of SFB. However, most existing studies on SFB fermentation only focus on the changes and correlations between microbial communities, physicochemical properties, and flavor substances, lacking a systematic analysis of the stage-specific microbial regulatory mechanisms of aldehyde metabolism and the mediating role of key physicochemical factors. This study focuses on investigating the microbial community structure, the dynamics of aldehyde compounds, and changes in physicochemical parameters at different stages of SFB pit fermentation. These investigations were conducted using high-throughput sequencing, GC-MS flavor analysis, physicochemical detection, and moisture regulation experiments, while simultaneously measuring the dynamic changes in core physicochemical indicators (such as moisture, temperature, starch, reducing sugars, etc.) and major aldehyde components in the fermented grains. Finally, the relevant regulatory mechanisms between microbial communities, physicochemical parameters, and aldehyde metabolism in the fermented grains were revealed through multivariate statistical analysis.

## 2. Materials and Methods

### 2.1. Sample Collection

The samples utilized in the test were gathered from the seventh-round fermented grains in pits of a SFB company located in Luzhou City, Sichuan Province, China. During the fermentation process of three pits, samples of pit-fermented grains were collected from 36 sites in the upper (D), middle (Z), and lower (X) layers on day 0 (D0), day 7 (D7), day 20 (D20), and day 30 (D30). Each layer is approximately one-third the depth of the cellar. The specific depth and orientation are shown in Figure 1A. After the samples from the five sampling points on the same layer were collected, they were immediately placed in sterile sealed bags and gently mixed to obtain the mixed sample of this layer. After the three layers of samples were processed separately, the same weight was drawn from the mixed samples of each layer and combined to form the final sample to be tested for this cell [26]. The physicochemical parameter-related samples were stored at −20 °C. Samples intended for microbial genomic DNA extraction and metabolome detection were treated with liquid nitrogen and then stored at −80 °C.

### 2.2. Environmental and Physicochemical Factors Determination

The temperature throughout the pit fermentation process was recorded. For the determination of alcohol content, the volatile substances in the sample were removed via distillation, after which the alcohol volume fraction in the Zaopei was measured using the alcohol meter method [27]. The acidity content was determined by the acid-base titration method, the moisture content by the constant-temperature drying method, by drying grains at 115 °C, and the reducing sugar and starch contents were determined using the 3,5-dinitrosalicylic acid (DNS) method [16].

### 2.3. Flavor Analysis

Sample pretreatment: 4 g sample was accurately weighed and mixed with 10 mL saline in a centrifuge tube, soaked overnight, then sonicated for 30 min and centrifuged at 10,000× *g* for 20 min. Five mL of the supernatant was transferred into a 20 mL headspace vial, then 20 µL pentane acetate (0.12 mg/100 mL) was added as the internal standard, along with 1.5 g sodium chloride. Extraction and determination of volatile flavor substances: the extraction head was inserted into the top empty bottle, equilibrated at 50 °C for 5 min, and then extracted at 50 °C for 45 min. Subsequently, in the no-splitting mode, the volatiles from the solid-phase microextraction (50/30 μm DVB/CAR/PDMS fiber) were removed at 230 °C for 5 min. Compound separation was performed on a Db-5 ms column (30 M 250 µM, 0.25 µM membrane thick; Agilent, Santa Clara, CA, USA). Helium gas (99.9995% purity) was used as a carrier gas at a constant flow rate of 1.0 mL/min. The column temperature program was set at 45 °C for 3 min, then increased to 150 °C at a rate of 4 °C/min for 2 min, and finally increased to 230 °C at a rate of 6 °C/min for 10 min. The ionization energy of the electron impact MS spectrum of 70 eV yielded 35–400 amu in the *m*/*z* scan range. The relative concentration of flavor substances was calculated based on the ratio of the peak area of the internal standard and the peak area of volatile metabolites.

### 2.4. Analysis of the Microbial Community

The V3–V4 region of the bacterial 16S rRNA gene and the internal transcriptional interval (ITS 1) region of the fungal 18S rRNA gene were amplified using the gene primers 338f/806r (5′-ACTCCTACGGGAGGCAGCAGAG-30/50-GGACTACHVGGTWTCTAAT-3′) and ITS1f/2043R (5′-CTTGGTCATTAGGAAGA-3′/5′-GCTGCGTTCTTCATCGATGC-3′). Extract and purify the DNA from Daqu using the E.Z.N.A.^®^ Soil DNA Kit (Omega Biotek, Norcross, GA, USA), following the detailed steps as outlined in the manufacturer’s instructions. The PCR amplification procedure was pre-denaturation for 3 min at 95 °C, 27 cycles (95 °C denaturation 30 s, 55 °C annealing 30 s, 72 °C extension 45 s), stable extension at 72 °C for 10 min and 10 °C until the reaction was stopped. The amplification system for bacteria was 4 μL, 5 FastPfu buffer, 2 μL dNTPs (2.5 mmol/L), 0.8 μL upstream primer (5 μmol/L), 0.8 μL downstream primer (5 μmol/L), 0.4 μL FastPfu DNA polymerase, 0.2 μL bovine serum albumin, 10 ng template DNA, ddH_2_O made up to 20 μL. The fungal amplification system was 2 μL 10 FastPfu buffer, 2 μL dNTPs (2.5 mmol/L), 0.8 μL upstream primer (5 μmol/L), 0.8 μL downstream primer (5 μmol/L), 0.2 μL rTaq polymerase, 0.2 μL bovine serum albumin, 10 ng template DNA, ddH_2_O made up to 20 μL. The same sample was extracted from DNA three times in parallel, and the purity and concentration of DNA were judged by NanoDrop8000 ultra-trace spectrophotometer, and qualified DNA samples were sent to Shanghai Major Biomedical Technology Co., Ltd. (Shanghai, China), for amplicon two-end (Paired-end) sequencing analysis of microbial sequences using the NovaSeq PE 250 platform of Illumina Company (San Diego, CA, USA).

### 2.5. Environmental Regulation During Pit Fermentation

To explore the impacts on the fermentation process, various moisture control strategies were adopted. In this experimental design, the moisture content of the fermented grains was adjusted to four distinct levels: 51.0% (designated as A), 51.5% (B), 52.0% (C), and 52.5% (D). Specifically, we achieved the goal of controlling the initial moisture content of the fermented grains by adding Hot Blending Water. This involved pouring hot water above 90 °C onto the grains. Moreover, a gradient adjustment of ±50 kg was implemented based on the standard dosage of hot blending water used in routine production. Meanwhile, all other operational parameters were kept constant. Samples were then gathered from three vertical locations-the upper, middle, and lower layers-within each fermentation pit. These samples were collected at two specific time points: day 0 (D0) and day 30 (D30), in preparation for subsequent analysis.

### 2.6. Statistical Analysis

All samples were tested in 3 replicates and the results were expressed as mean ± standard deviation. The α index was calculated by mothur software [28], and the physical and chemical data were processed and graphed by Origin 2022 (Origin Lab, Northampton, MA, USA). Redundancy analysis/Canonical correspondence analysis (RDA/CCA) was used to analyze the correlation between environmental variables and microbial communities. The microbial data processing was the same as 3.2. Visualized correlation data painting was performed using the Gephi software (version 0.9.2). Based on Spearman correlation coefficient, the correlation between microorganisms and metabolites and microorganisms and chemical factors was analyzed by R (4.2.2).

## 3. Results

### 3.1. Physicochemical Factors in the Process of Pit Fermentation

The pit environment plays a pivotal role in the development of Baijiu flavor. Alterations in the pit’s physicochemical conditions, microorganisms, and metabolic products have a more direct bearing on the final flavor of Baijiu [29]. As fermentation unfolds, the pit environment undergoes continuous transformation. To delve into the factors influencing microorganisms associated with aldehyde synthesis, we measured the physicochemical properties of the pit to explore their patterns of change (Figure 1B). As depicted in the figure, as fermentation time elapses, the temperature initially rises, then stabilizes, and ultimately declines. The pH exhibits a slightly downward trend, while the total acid content increases steadily, signifying the continuous formation and accumulation of organic acids and other acidic substances. The moisture content generally increases over the course of fermentation due to the reproduction [30]. Starch and reducing sugars decrease overall because microorganisms need energy, and these substances serve as their main energy sources. The ethanol content rises continuously with the progression of fermentation time [31]. The deceleration in ethanol production towards the end of the fermentation process can be attributed to several factors: exhaustion of fermentable sugars, an enhanced inhibitory effect of ethanol, and acid stress. By analyzing these physical and chemical factors, it is clear that the environment formed by the increase in total acid and ethanol content as the fermentation time progresses will force substantial changes in the microbial community structure [32].

### 3.2. Microbial Community Richness and Diversity During Pit Fermentation

The bacterial representative sequences were taxonomically annotated via the Silva138 database, and the fungal sequences were annotated through the UNITE8.0 database, both using the classify-sklearn classifier. For the purpose of facilitating downstream diversity and composition analyses, sequence rarefaction was carried out. This was based on the minimum sample sequence count, with bacterial sequences rarefied to 1676 sequences per sample and fungal sequences rarefied to 13,272 sequences per sample. Ultimately, 60,336 bacterial sequences along with 3171 Amplicon Sequence Variants (ASVs) were acquired. Simultaneously, 477,792 fungal sequences and 4463 ASVs were obtained. As depicted in the Sobs index rarefaction curves (Figure S1), when the sample size grew large enough, the rarefaction curves gradually flattened out. This indicates that the sequencing depth was sufficient to comprehensively represent the microbial communities within all samples. In other words, this sequencing captured the vast majority of microbial species information [33].

The α-diversity indices of microbial communities are commonly manifested through various metrics like Chao1 and Shannon. The Chao1 index places an emphasis on community richness, whereas the Shannon index reflects community diversity [34]. The microbial α-diversity indices were computed, and one-way ANOVA was employed to compare the α-diversity indices of microorganisms in the pit at different time points (Figure 1C). The findings revealed that there was no substantial alteration in bacterial richness from day 7 to day 20. However, a significant disparity was detected from day 20 to day 30, signifying that bacterial richness decreased significantly after 20 days. Regarding bacterial diversity, no significant differences were noted from day 0 to day 7 and from day 20 to day 30. Nevertheless, a highly significant difference was observed from day 7 to day 20. This implies that bacterial diversity declined rapidly after 7 days and remained stable post-20 days. Likewise, fungal richness increased significantly on day 7 and remained stable after 20 days. Fungal diversity, on the other hand, gradually increased from day 0 to day 20 and then leveled off. Bacterial species richness initially rose and then fell. Notably, from day 7 to day 20, the changes in temperature and nutrients such as starch were not drastic. This indicates that species richness should not have changed markedly during this period. It suggests that bacterial richness reached its peak around day 7, while fungal richness peaked around day 20.

By observing the physical and chemical environment within the pit, it is evident that as fermentation progresses, the pit environment undergoes substantial changes. The increasing levels of total acid and ethanol gradually render the environment more inhospitable. This inevitably exerts a significant influence on the structure and composition of the microbial community. When considering the earlier discussion regarding the pattern of microbial community succession, we have discovered the following: In the initial fermentation stage, a relatively higher pH value, along with abundant nutrients like starch and reducing sugars, may be more favorable for the growth and reproduction of bacteria. Subsequently, as the environment gradually changes. For instance, when the acidity rises and ethanol accumulates, this may suppress the activity of bacteria and instead help the fungal community thrive. This finding aligns with the previous analysis of acidity and ethanol levels.

### 3.3. Microbial Composition and Succession of the Pit Fermentation Process

**Figure 2:**
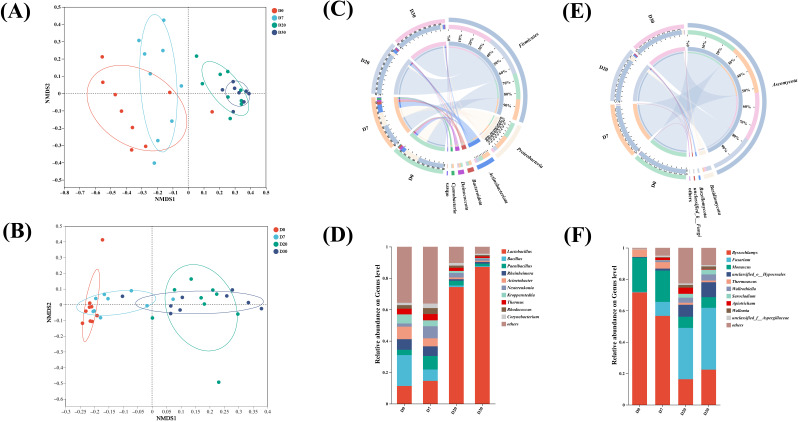
NMDS analysis results of bacteria (**A**) and fungi (**B**) during the fermentation process in the cellar. The distributions of bacteria at the phylum level (**C**) and the genus level (**D**). The distributions of fungi at the phylum level (**E**) and the genus level (**F**) (*p* < 0.05).

To delve deeper into the stage-specific traits of microbial communities throughout the fermentation process, Non-Metric Multidimensional Scaling (NMDS) analysis was conducted on the microorganisms (Figure 2A,B) [35]. In the NMDS plot, the samples from days 0 and 7 are closely grouped together. Meanwhile, the samples from days 20 and 30 form a separate, tightly knit cluster. This suggests that, in terms of community structure composition, the samples within the 0–7-day range are highly alike, and the samples within the 20–30-day range are also highly similar to one another. However, there are marked differences between these two sets of samples. Samples from the 7–20-day period are precisely positioned in the “transition zone” between these two stable clusters on the NMDS map. Their community structure is in a state of continuous and rapid change and has not yet formed a stable, independent cluster. Consequently, bacteria and fungi can be clearly divided into two parts. Specifically, days 0–7 of the in-pit fermentation are categorized together (the early stage of in-pit fermentation), while days 20–30 are grouped as another part (the late stage of in-pit fermentation). Evidently, considering the stage-specific characteristics of microorganisms, the in-pit fermentation process can be bifurcated into an early stage and a late stage.

In the analysis of community diversity throughout the entire fermentation process, the main bacterial phyla (Figure 2C) include *Firmicutes*, *Proteobacteria*, *Actinobacteriota*, and *Cyanobacteria*. Among these, *Firmicutes* is the dominant bacterial phylum. The main bacterial genera (Figure 2D) are *Lactobacillus*, *Bacillus*, *Paenibacillus*, *Rheinheimera*, and *Acinetobacter*. Initially, the abundance of lactic acid bacteria (with *Lactobacillus* being a key lactic-acid-producing genus) accounted for only 11.3% in the early stage of fermentation. However, as fermentation progressed, lactic acid bacteria gradually became the dominant genus, reaching 87.1% in the later period. Intriguingly, findings from other studies also show that, in most cases, *Lactobacillus* has the tendency to replace other genera and become the dominant genus [36,37]. Bacillus, on the other hand, was the dominant bacterium in the early stage. But as fermentation time elapsed, it appeared that Bacillus could not adapt well to the pit environment. As a result, it gradually vanished, with its abundance dropping to only 0.5% in the later stage of fermentation.

The fungal phyla (Figure 2E) mainly consist of *Ascomycota*, *unclassified_k_Fung*, *Rozellomycota*, and *Basidiomycota*. Among these, *Ascomycota* has remained the dominant phylum because of its robust viability and adaptability. The main fungal genera (Figure 2F) include *Byssochlamys*, *Fusarium*, *Monascus*, *unclassified_o_Hypocreales*, and *Thermoascus*. It was observed that *Byssochlamys* (accounting for 71.6%) and *Monascus* (21.8%) were the dominant fungi in the early stage of fermentation. However, as fermentation progressed, their abundances first decreased and then increased, yet the overall tendency was a decline. It is hypothesized that organic acids and certain metabolites altered the micro-environment within the pit, making it challenging for some fungi to thrive [38]. Conversely, *Fusarium* and *unclassified_o_Hypocreales* gradually increased in abundance as fermentation advanced, suggesting that they could adapt to the environmental changes in the later stages. By the late stage of fermentation, the dominant fungal genus was Fusarium, which accounted for 39.4%.

### 3.4. Dynamic Changes in Aldehyde Compounds During Pit Fermentation

**Figure 3:**
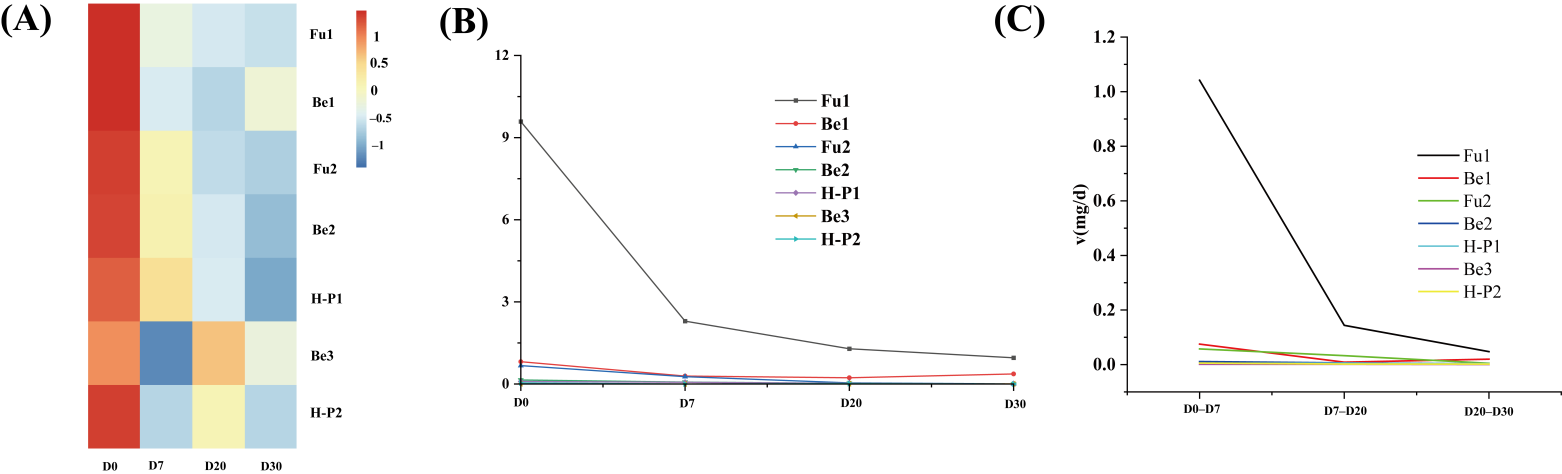
(**A**) Correlation heatmap of aldehydes during pit fermentation. (**B**) Changes in aldehyde compounds over fermentation days. (**C**) Comparison of aldehyde consumption rates (Fu1 = Furfural, Fu2 = 5-methylfuran aldehyde, Be1 = Benzaldehyde, Be2 = Phenylacetaldehyde, Be3 = Benzeneacetaldehyde, alpha-ethylidene-, H-P1 = 1H-Pyrrole-2-carboxaldehyde, H-P2 = 1-methyl, 1H-Pyrrole-2-carboxaldehyde).

During the fermentation process of the SFB pit pool, the dynamic changes in aldehyde compounds, which are important flavor substances, and their association mechanisms with microbial metabolic activities remain incompletely understood. To address this, we employed the GC-MS combined technology to detect and quantify the flavor composition of the pit-fermented grains (Supplementary Material). As a result, seven major aldehyde compounds were identified, namely Furfural (Fu1), Benzaldehyde (Be1), 5-methylfuran aldehyde (Fu2), Phenylacetaldehyde (Be2), Benzeneacetaldehyde, alpha-ethylidene-(Be3), 1H-Pyrrole-2-carboxaldehyde (H-P1), and 1-methyl, 1H-Pyrrole-2-carboxaldehyde (H-P2) (Figure 3A). Furfural is the dominant aldehyde throughout the fermentation process (Figure 3B), constituting over 70% of the total aldehyde content. This is especially prominent on day 0, where it accounts for 84.41% of the total aldehyde content. Despite some variations in the absolute content of aldehyde compounds, their dynamic change trends are highly consistent, showing a pattern of continuous decline. Further analysis (Figure 3C) revealed that the consumption rate of aldehydes in the first 7 days was significantly higher than that in the subsequent 20-day period. It is speculated that there might be specific conditions for aldehyde degradation in the early stage of fermentation. This pattern of change suggests that aldehyde compounds were rapidly consumed when the fermented grains were first placed in the pit. During the subsequent fermentation process, aldehyde compounds were gradually either consumed or transformed. In the early stage of SFB fermentation, due to the presence of a certain amount of available oxygen in the pit, some aerobic microorganisms could maintain high metabolic activity [39,40]. Additionally, in this stage, there are ample reserves of sugar compounds and other nutrients, which serve as a rich substrate and energy source for microbial metabolism. In this micro-aerobic environment, aldehydes are likely to be further oxidized by aerobic microorganisms to produce corresponding metabolites [41,42]. Consequently, in the early stage of fermentation, the brief microaerobic conditions and abundant nutrients create a suitable environment for the vigorous metabolic activities of aerobic microorganisms. This leads to the rapid degradation and oxidation of aldehyde compounds, resulting in a high consumption rate. As the fermentation process progresses, the environment gradually turns into a strictly hypoxic state. The activity of aerobic microorganisms is inhibited, and the consumption rate of aldehyde compounds also drops [36].

### 3.5. Relationship Between Microorganisms and the Aldehyde of the Pit Fermentation Process

**Figure 4:**
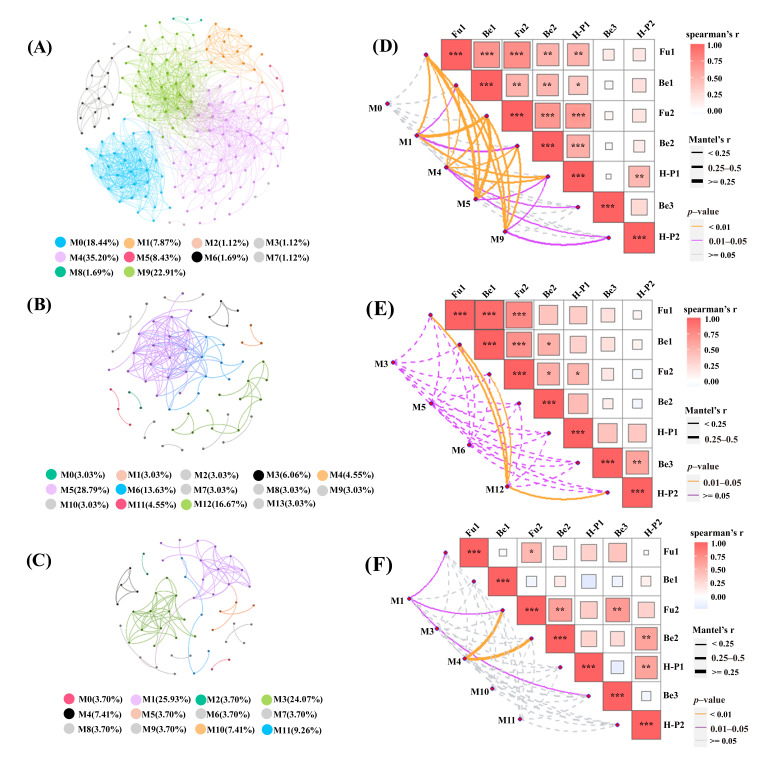
Co-occurrence network analysis of the fermentation process in the cellar (r > 0.6, *p* < 0.001), and the microbial co-occurrence network was colored by module (* *p* < 0.05, ** *p* < 0.01, and *** *p* < 0.001). (**A**) Co-occurrence network from day 0 to day 30; (**B**) Co-occurrence network from day 0 to day 7; (**C**) Co-occurrence network on the 20th to 30th day; (**D**) Mantel test for microbial networks and aldehydes during the fermentation process from day 0 to day 30; (**E**) Mantel test for microbial networks and aldehydes during the fermentation process from day 0 to day 7; (**F**) Mantel test for microbial networks and aldehydes during the fermentation process on the 20th to 30th day.

To explore the relationship between aldehyde compounds and microbial metabolism, genus-level co-occurrence networks were analyzed for the time periods of days 0–30 (Figure 4A), days 0–7 (Figure 4B), and days 20–30 (Figure 4C). Looking at the overall pit fermentation, as depicted in Figure 4A, in the co-linearity network model of fermentation microbes within the cellar, there are a total of 178 nodes and 1091 edges. The entire co-linearity network model is divided into 10 modules. Among these, the modules with a proportion greater than 5% are module M0 (18.44%), module M1 (7.87%), module M4 (35.20%), module M5 (8.43%), and module M9 (22.91%). Module M4 is the largest module, having 63 nodes, which include 56 bacterial genera and 7 fungal genera. Module M9 is the second-largest module, with 41 nodes, consisting of 1 bacterial genus and 40 fungal genera. Modules M1 and M5 have 14 and 15 nodes, respectively. Module M1 contains 14 bacterial genera, and module M5 contains 9 bacterial genera and 6 fungal genera. Based on the NMDS analysis and co-occurrence networks analysis, the co-occurrence network for days 0–7 (the early fermentation stage) is composed of 66 nodes and 151 edges. All nodes in this co-occurrence network are grouped into 14 modules. The modules that exceed 5% are module M3 (6.06%), module M5 (28.79%), module M6 (13.63%), and module M12 (16.67%). Module M12 is the second-largest module, with 12 nodes, including 4 bacterial genera and 8 fungal genera. The co-occurrence network for days 20–30 (the late fermentation stage) consists of 54 nodes and 95 edges. All nodes in this network are clustered into 12 modules. The modules exceeding 5% are module M1 (25.93%), module M3 (24.07%), module M4 (7.41%), module M10 (7.41%), and module M11 (9.26%). Modules M1 and M4 have 14 and 4 nodes, respectively. Module M1 contains 11 bacterial genera and 3 fungal genera, while module M4 contains 3 bacterial genera and 1 fungal genus. Compared to the late fermentation stage, the network structure in the early stage is more complex. This indicates that the interactions from days 0–7 are stronger than those in the network structure from days 20–30, suggesting a more active metabolic level during the early fermentation stage.

Co—linearity network analysis and correlation analysis were conducted. These analyses were carried out for microorganisms and aldehydes throughout the entire in pit fermentation process (Figure 4D), as well as during the early (Figure 4E) and late (Figure 4F) fermentation stages. As a result, the relationship between microorganisms and aldehyde variations was elucidated. Aldehyde compounds tend to decline as the fermentation time progresses. The correlations among aldehyde compounds are predominantly positive, suggesting that most aldehydes are metabolized into other substances by microorganisms. Regarding the entire fermentation process, module M0 exhibited no significant correlation with aldehyde compound synthesis. Module M1 was significantly correlated with the changes in Fu1, Fu2, and H.P1. Module M4 was significantly associated with the changes in Fu1, Be1, Be2, Fu2, and H.P1. Module M9 was significantly correlated with the changes in Fu1, Be1, Be2, and Fu2. However, during the early fermentation stage, only module M12 demonstrated a significant correlation with the changes in Fu1, Be1, Fu2, and H.P2, while other modules showed no such correlation. In the late fermentation stage, module M1 was correlated with Fu1 and Fu2, and module M4 showed a significant correlation with Fu2 and Be2. This implies that the dynamic changes in aldehyde compounds are likely not a result of simple chemical oxidation-reduction reactions. Instead, they are regulated by microbial metabolic activities. The microorganisms within these four modules are linked to most aldehyde compounds, indicating that the microorganisms in these modules may either promote or inhibit the formation or degradation of these aldehydes.

According to the degree centrality, closeness centrality, and betweenness centrality data in each module, the key microbes are extracted. For the whole fermentation cycle, in module M1, the bacterial genus *Paenibacillus*; in module M4, the fungal genus *Virgibacillus, Stenotrophomonas*, the bacterial genus *Clostridium_sensu_stricto_3*, *Bacillus, Sanguibacter, Lactobacillus*; in module M5, the fungal genus *Apiotrichum*; in module M9, the fungal genera *Fusarium, Wallrothiella, Cladosporium, Exophiala*, and *unclassified_f_Trichosphaeriaceae* are the key microbes in these modules. In the early fermentation stage, in module M12, the fungal genus *Fusarium, Apiotrichum_scarabaeorum*. In the late fermentation stage, in module M1, the bacterial genus *Bacillus, Paenibacillus_pasadenensis*; in module M4, the bacterial genus *unclassified_c_Bacilli, Lactobacillus_acetotolerans*, the fungal genus *Sclerostagonospora_rosae*. This indicates that during the early stages of the fermentation process, changes in aldehyde compounds are primarily influenced by fungi. However, as the fermentation progresses, both bacteria and fungi jointly affect the changes in aldehyde compounds.

The fermentation process shows clear differences in microbial dynamics between the early and late stages, which have diverse effects on aldehyde metabolism. Our study has uncovered that there is a coordinated transformation of aldehyde compounds into other metabolites, mediated by microorganisms [43]. This is manifested by the decline in aldehyde concentrations over time and the mostly positive correlations among these compounds. Significantly, certain microbial modules demonstrated stage-specific correlations with aldehyde compounds. This indicates that as fermentation advances, there is a temporal change in how microorganisms influence aldehyde metabolism. The complex interaction between microorganisms and aldehyde compounds implies that the dynamics of aldehydes are mainly regulated by microbial metabolic activities, rather than being the result of simple chemical reactions. Identifying the key microbial modules related to multiple aldehyde compounds clarifies the complex relationships between microbial communities and the evolution of flavor compounds. Moreover, it offers potential focal points for future research into the mechanisms of aldehyde formation and degradation during fermentation.

### 3.6. The Relationship Between Physicochemical Environment and Microorganisms Related with Aldehyde Synthesis

**Figure 5:**
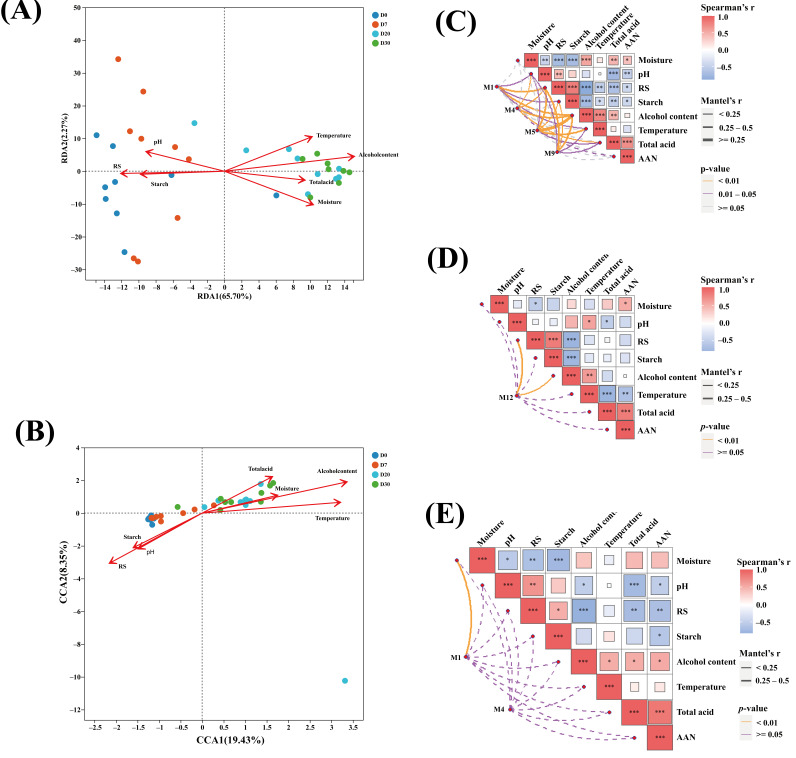
Redundancy analysis was conducted between bacteria (**A**) and environmental variables, and canonical correlation analysis was carried out between fungi (**B**) and environmental variables during pit fermentation. A Mantel test was performed between microbial networks and environmental variables during fermentation (* *p* < 0.05, ** *p* < 0.01, and *** *p* < 0.001). The Mantel test for days 0–30 (**C**). The Mantel test for days 0–7 (**D**). The Mantel test for days 20–30 (**E**).

The potential association between microorganisms and physicochemical parameters during pit fermentation was examined using Redundancy Analysis (RDA) or Canonical Correspondence Analysis (CCA). First, the appropriate model (RDA or CCA) was selected by analyzing the length of the first axis through Detrended Correspondence Analysis (DCA). The DCA results showed that the length of the first axis was 2.76 for bacteria and 4.58 for fungi. Generally, in DCA, when the length of the first axis is greater than 4.00, the RDA model is employed, and when it is less than 3.00, the CCA model is used [44]. Thus, the RDA model was used for bacteria (Figure 5A), and the CCA model was used for fungi (Figure 5B). As depicted in the figures, these two axes account for 70.78% and 27.57% of the variation in bacterial and fungal communities, respectively. In the early stage of pit fermentation, pH, starch, and reducing sugar are the key factors influencing microbial succession. In contrast, in the late stage of fermentation, the main influencing factors are total acid, temperature, ethanol, and moisture. The moisture content was found to have a significant negative correlation with reduced sugar and starch, yet a positive correlation with ethanol content. Starch was positively correlated with the reduced sugar content. The pH value was negatively correlated with total acidity. Ethanol synthesis was negatively correlated with starch and reduced sugar content but positively correlated with temperature. This suggests that a high temperature during the late fermentation stage provides a favorable condition for ethanol synthesis.

The dynamic alterations of aldehyde compounds are intricately linked to the metabolic activities of microorganisms during the fermentation process. Simultaneously, the growth and metabolism of these microorganisms are markedly influenced by the physicochemical conditions within the fermentation environment [45]. The study utilized the Mantel test to analyze the correlations between different microbial modules and environmental variables. The aim was to uncover the microbial communities associated with the patterns of aldehyde compound changes and the environmental factors driving them. For the entire fermentation cycle (Figure 5C), the findings indicated that module M1 (including bacteria of the genus *Paenibacillus*) had a significant correlation with ethanol and reducing sugars. Module M4 (featuring *Lactobacillus*, *Virgibacillus*, etc.) was closely associated with pH, total acidity, ethanol, and reducing sugars. Module M5 (such as the fungal genus *Apiotrichum*) was strongly connected to ethanol, temperature, and reducing sugars. Moreover, module M9 (including *Acinetobacter*, *Wallrothiella*, etc.) was highly correlated with reducing sugars, ethanol, and starch content. During the early fermentation stage (Figure 5D), module M12 (comprising *Fusarium*, *Apiotrichum_scarabaeorum*, etc.) was closely related to ethanol and reducing sugars. In the late fermentation stage (Figure 5E), module M1 (like *Bacillus*, *Paenibacillus_pasadenensis*, etc.) was closely associated with moisture, while M4 did not appear to have a correlation with physicochemical parameters. These results demonstrate that the dynamic changes in the physicochemical conditions of the fermentation environment directly impact the metabolic pathways and activities of microbial communities. Consequently, this regulates the biosynthesis and degradation processes of metabolic products, such as aldehyde flavor compounds.

**Figure 6:**
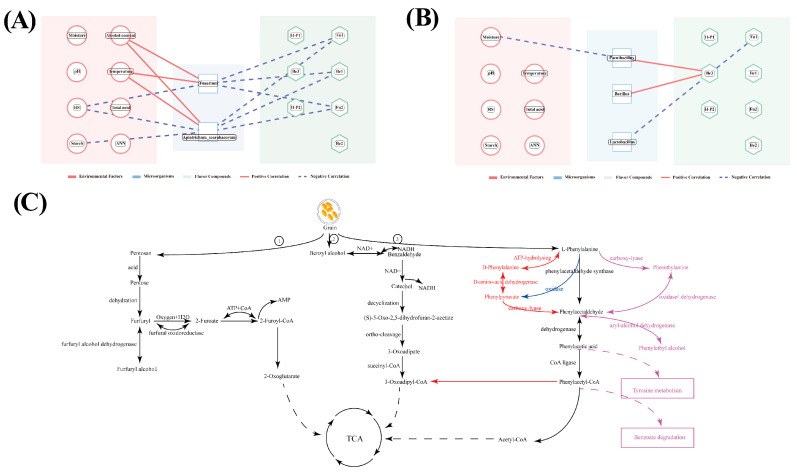
Environment–Microbe–Flavor Network Analysis during Pit Fermentation at 0–7 days (**A**) and 20–30 days (**B**). (*p* < 0.05, |r| > 0.5). The aldehydes metabolic profile is (1) the metabolic pathway of furfural, (2) the metabolic pathway of benzaldehyde, and (3) the metabolic pathway of phenacetaldehyde (**C**).

Through the Environmental–Microbe–Flavor Network analysis, the study delved into the dynamic interactions among environmental factors, microorganisms, and flavor compounds during in-cellar fermentation. In the early fermentation stage (Figure 6A), specific environmental conditions played a crucial role. Higher levels of ethanol and temperature, coupled with lower amounts of starch and reducing sugars, created an environment conducive to the growth of *Fusarium* and *Apiotrichum_scarabaeorum*. These microorganisms then consumed Fu1, Be1, and Fu2. This phenomenon can potentially account for the rapid decrease in aldehydes like furfural (Fu1) in the early stage. As for the late fermentation stage (Figure 6B), the relationships between physicochemical factors and certain bacteria, namely *Bacillus* and *Lactobacillus*, were less distinct. However, moisture was negatively correlated with *Paenibacillus*. At this point, *Paenibacillus* and *Bacillus* had a positive correlation with Be3, while *Lactobacillus* was negatively correlated with Fu1. These results are in line with previous findings, suggesting that the microbial community structure within the cellar and the aldehyde compounds display dynamic interactions and differences specific to each stage of fermentation. In the early stage, *Fusarium* and *Apiotrichum_scarabaeorum,* by utilizing available energy sources, quickly consume aldehydes such as Fu1. In the later stage, a state of dynamic stability is maintained.

To mechanistically interpret the microbially driven dynamic changes in aldehydes observed in this study, we integrated our research findings with potential metabolic pathways inferred from the KEGG database and relevant literature. The rapid consumption of aldehydes (especially in the early stage of fermentation) (Figure 3C) strongly indicates that the microbial community actively catabolizes them as carbon and energy sources. Our co-occurrence network and Mantel test analyses identified predictive microbial modules associated with these changes. For example, the early-stage Module M12, dominated by *Fusarium* and *Apiotrichum*, was significantly correlated with the degradation of furfural (Fu1) and benzaldehyde (Be1) (Figure 4D). This correlation coincided with a period of high microbial activity and available oxygen, supporting the hypothesis that these fungi may oxidize aldehydes. We put forward a likely metabolic pathway. Furfural undergoes oxidation to become furoic acid and has the potential to access central carbon metabolism by way of acetyl—CoA. Concurrently, benzaldehyde and phenylacetaldehyde (Be2) are assimilated separately through the microbial benzoate degradation pathway and the phenylalanine/tyrosine metabolism pathway. Eventually, they both enter the TCA cycle (Figure 6C). As fermentation progressed to the late stage, microbial dominants and environmental conditions underwent a shift. Under increasingly anaerobic and acidic conditions, the association of aldehydes with bacterial genera such as *Lactobacillus* and *Paenibacillus* (Modules M1 and M4, Figure 4F) suggests a distinct metabolic strategy. These bacteria may be involved in reducing aldehydes to their corresponding alcohols or other reductive pathways, which could explain the slower rate of change in aldehyde concentrations observed in the late stage of fermentation (Figure 3C). The significant negative correlation between moisture and *Paenibacillus* further indicates that environmental factors shape the interactions of these late-stage microorganisms and their associated aldehyde metabolism. Ultimately, the depletion of fermentable substrates such as starch in the late stage may limit the de novo synthesis of aldehydes, leading to their net decline. Although this study establishes functional links between specific microbial communities and aldehyde dynamics, the specific enzymatic pathways employed by key genera (e.g., *Lactobacillus* and *Fusarium*) (e.g., the role of aldehyde dehydrogenases or reductases) remain to be validated. Future work employing metatranscriptomics to analyze gene expression, combined with targeted metabolomics to track aldehyde metabolic fluxes, will be crucial for elucidating the precise molecular mechanisms underlying this key aspect of SFB fermentation.

### 3.7. Environmental Regulation During Pit Fermentation

**Figure 7:**
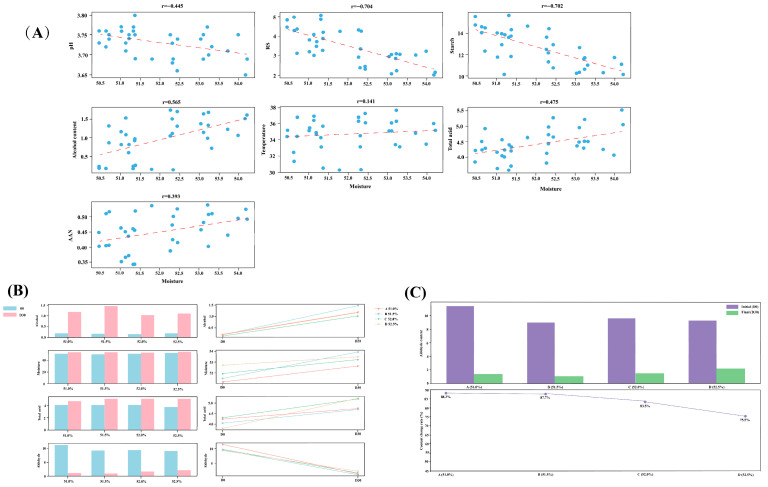
(**A**) Correlation analysis between moisture and other physicochemical parameters during pit fermentation (*p* < 0.05). (**B**) Changes in physicochemical parameters during moisture regulation. (**C**) Evolution of aldehyde content under different moisture regulation conditions. Note: The blue circle represents the physicochemical properties of each sample under the corresponding moisture during the fermentation process, and the virtual shadow represents the trend line.

The relationships between moisture and other physicochemical parameters demonstrated substantial interactions occurring throughout the pit fermentation process (Figure 7A). Moisture had a robust negative correlation with reducing sugar (with a correlation coefficient r = −0.704) and starch (r = −0.702). This indicates that an increase in moisture content could potentially speed up the consumption of these substrates. Furthermore, a moderately positive correlation was detected between moisture and alcohol content (r = 0.565). This implies that moisture might have an important part to play in the formation of alcohol. Regarding the other parameters, namely pH, temperature, total acid, and AAN (amino acid nitrogen, assuming), they had weak to moderate correlations (∣r∣ < 0.5) with moisture. Collectively, these correlations highlight that the moisture content is of great significance in the utilization of substrates and the formation of metabolites during fermentation. As a result, this finding motivated us to explore the impacts of regulating moisture on the outcomes of the fermentation process.

Building on the previous findings, moisture regulation experiments were carried out. The initial moisture content was controlled at four levels: 51.0% (A), 51.5% (B), 52.0% (C), and 52.5% (D). The physicochemical analysis (Figure 7B) uncovered distinct trends in different moisture treatments during fermentation. For alcohol content, the B treatment witnessed the most substantial increase, rising from 0.17% to 1.47%. However, as the moisture content increased, the final alcohol contents decreased, reaching 1.13% in the C treatment and 1.14% in the D treatment. Regarding total acid, it increased in all treatments, with the C and D groups showing more notable increases. In the C group, it rose from 4.29 to 5.21, and in the D group, from 3.79 to 5.23. The analysis of aldehyde evolution (Figure 7C) indicated that the aldehyde content decreased significantly across all treatments. Starting from initial values ranging from 9.27–11.36, it dropped to final values of 1.10–2.27. The A treatment showed the most effective degradation, with an 88.3% decrease, closely followed by the B treatment with an 87.7% decrease. The treatment with the highest moisture content (D, 52.5%) had the lowest degradation rate, at 75.5%. This degradation pattern is consistent with the previously identified negative correlations between moisture and reducing sugar (r = −0.704) and starch (r = −0.702), suggesting that the moisture content has a significant impact on substrate utilization. Based on the physicochemical analysis, within the tested range of 51.0–52.5%, controlling the initial moisture content at 51.0–51.5% demonstrated the optimal aldehyde degradation efficiency. These results highlight that proper moisture control is essential for aldehyde transformation during fermentation. Nevertheless, the specific mechanism underlying this phenomenon requires further investigation, which could involve a combination of metabolomics and microbial genomic analysis.

## 4. Discussion

This study systematically reveals the stage—specific microbial regulatory mechanism of aldehyde metabolism during the pit fermentation of SFB. It also identifies moisture as the core environmental regulatory factor. This provides a new perspective for understanding the microbial—driven mechanism of flavor formation and optimizing the fermentation process of SFB. Based on microbial succession patterns, the pit fermentation of SFB can be clearly divided into two stages: the early stage (0–7 d) and the late stage (20–30 d), and this stage division directly drives the dynamic changes in aldehyde compounds. This result is consistent with previous research conclusions on microbial community succession during SFB fermentation, but further establishes a direct causal relationship between “stage-specific microorganisms and aldehyde metabolism”, making up for the deficiencies of previous studies.

In the early stage of fermentation, *Fusarium* and *Apiotrichum scarabaeorum* dominate. Combined with increased ethanol content, elevated temperature, and sufficient substrates, they collectively lead to the rapid metabolic transformation of aldehyde compounds. This finding supplements the existing cognition that “fungi are key taxa for flavor metabolism in the early stage of fermentation”, and further clarifies that these two genera are the core regulatory microorganisms for aldehyde transformation. Notably, Redundancy Analysis (RDA) indicates that ethanol and temperature have a positive correlation with the growth of Fusarium. This suggests that the initial microaerobic environment maintained in the pit in the early stage might increase the metabolic activity of these aerobic fungi, facilitating the oxidation of aldehydes to acids or esters. This pathway is consistent with KEGG-inferred aldehyde metabolism pathways.

In the late stage of fermentation, the community dominance shifts to *Paenibacillus* and *Lactobacillus*, and moisture becomes the primary regulatory factor. Moisture is significantly negatively correlated with *Paenibacillus* (r = −0.68, *p* < 0.05), indicating that excessive moisture may inhibit the transformation ability of this genus on aldehydes (such as Be3, alpha-ethylidene-benzeneacetaldehyde). This forms a sharp contrast with the “temperature and ethanol-dominated regulation” in the early stage, highlighting the temporal dynamics of environmental driving factors for aldehyde metabolism, a phenomenon less commonly addressed in previous studies on SFB fermentation.

This study confirms that moisture is a key environmental factor regulating substrate utilization and aldehyde transformation. When the initial moisture content is 51.0–51.5%, the aldehyde degradation efficiency is optimal (87.7–88.3%); when the moisture content increases to 52.5%, the degradation efficiency decreases to 75.5%. This finding fills the gap in practical brewing production. Traditional SFB fermentation mostly relies on empirical moisture control, lacking scientific basis for optimal parameters.

The mechanism by which moisture regulates aldehyde metabolism is reflected in its dual effects on microbial communities and substrate availability. As shown in Figure 7A, moisture is strongly negatively correlated with starch and reducing sugars. This indicates that excessive moisture will accelerate substrate consumption, exhausting the energy source of aldehyde—regulating microorganisms (such as *Fusarium* in the early stage) and limiting the regeneration of aldehydes in the late stage. Meanwhile, high moisture may reduce the oxygen diffusion efficiency in the pit, favoring the growth of anaerobic microorganisms (such as *Lactobacillus*). Figure 6B shows that *Lactobacillus* is negatively correlated with furfural, indicating that such bacteria have weak aldehyde degradation ability, and their dominant growth will further weaken the overall aldehyde metabolism efficiency.

This study briefly clarifies the intrinsic correlation logic of “stage—microorganism—aldehyde metabolism—environmental factor” during the pit fermentation of SFB. It also proposes a process optimization scheme directly applicable to production practice, like the moisture control standard of 51.0–51.5% in the seventh fermentation cycle.

This study contributes to the scientific understanding of the flavor formation mechanism of traditional solid—state fermented foods. It provides a specific case for analyzing the process of microbial and environmental factors synergistically regulating flavor metabolism and can directly guide the parameter optimization at the production end. In addition, this study lays a foundation for subsequent research on parameter regulation strategies for other fermentation cycles or the analysis of key mechanisms of aldehyde metabolism using multi-omics technologies.

## 5. Conclusions

This study systematically clarifies the “stage–microorganism–environmental factor” synergistic regulatory network of aldehyde metabolism during the pit fermentation of SFB. It further refines the mechanism by which *Fusarium* and *Apiotrichum scarabaeorum* achieve rapid aldehyde transformation relying on ethanol accumulation and temperature elevation under the microaerobic environment in the early stage. Meanwhile, in the late stage, moisture becomes the key environmental factor regulating aldehyde metabolism efficiency by inhibiting the activity of *Paenibacillus* and promoting the proliferation of *Lactobacillus* with weak aldehyde degradation ability. The established initial moisture content of 51.0–51.5% for the seventh fermentation cycle converts the empirical moisture control in traditional brewing into quantifiable scientific parameters. This effectively avoids the pungent odor caused by excessive aldehydes, improves the flavor consistency of base liquor batches, and provides a key reference for the research on moisture parameter adaptation in other fermentation cycles of SFB.

## Data Availability

The original contributions presented in the study are included in the article/Supplementary Materials. Further inquiries can be directed to the corresponding authors.

## Supplementary Materials

The following supporting information can be downloaded at: https://www.mdpi.com/article/10.3390/foods15010017/s1, Figure S1: Dilution curves of microbial community in fermented grains (a) dilution curve of fungi in the cellar; (b) dilution curve of bacteriain the cellar. Excel contains the flavor data of all samples. Each worksheet represents one sample, and the naming principle is round + cellar number + fermentation time + spatial location. For instance, SD7M2D7D: Top sample on the 7th day of fermentation in the second cellar of the seventh round.
