# Supplementary material for: Process Regulation of Microbial-Driven Aldehyde Metabolism in Sauce-Flavor Baijiu Fermentation
Source: Foods. 2025 Dec 21;15(1):17. doi: 10.3390/foods15010017 (PMC12785534; doi:10.3390/foods15010017)
Supplement: Supplementary file 1 [file foods-15-00017-s001.zip › Supplementary_Material.pdf]

foods

Supplementary Material

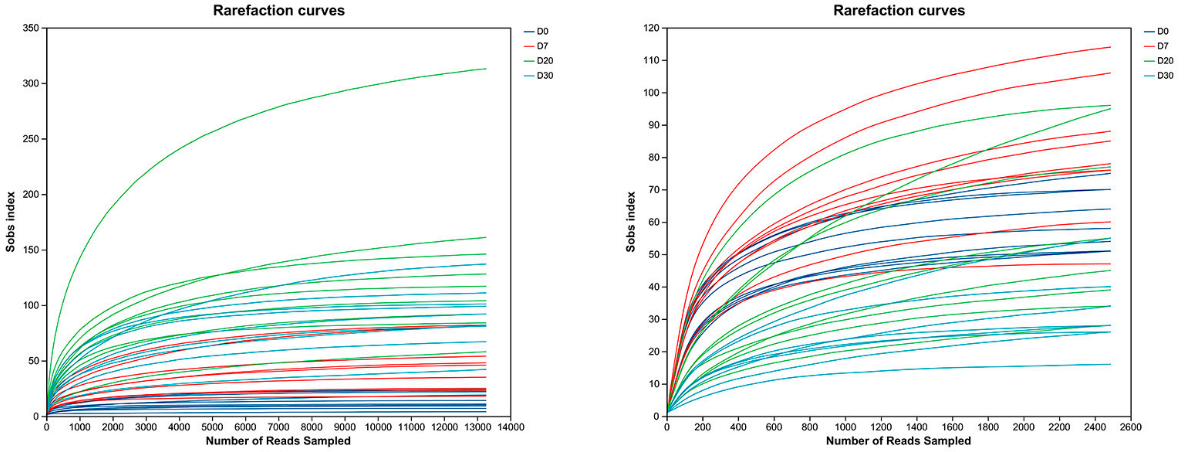

(a) (b)

Supplementary Figure S1. Dilution curves of microbial community in fermented grains(a) dilution curve of fungi in the cellar ; (b) dilution curve of bacteriain the cellar.
